# Analysis of Polarization Images in the Microphysical Blood Parameters Research for the Hematocrit Diagnostics

**DOI:** 10.3390/mi13122241

**Published:** 2022-12-16

**Authors:** Ruslan D. Khlynov, Victoria A. Ryzhova, Sergey N. Yarishev, Igor A. Konyakhin, Valery V. Korotaev, Yuri E. Shelepin, Todor S. Djamiykov, Marin B. Marinov

**Affiliations:** 1 Applied Optic Centre, ITMO University, Kronverksky Pr. 49, Bldg. A, 197101 St. Petersburg, Russia; 2 School of Physics and Engineering, ITMO University, Kronverksky Pr. 49, Bldg. A, 197101 St. Petersburg, Russia; 3 Higher School of Engineering and Technology, ITMO University, Kronverksky Pr. 49, Bldg. A, 197101 St. Petersburg, Russia; 4 Pavlov Institute of Physiology, Russian Academy of Sciences, Makarova Embankment, 6, 199034 St. Petersburg, Russia; 5 Department of Electronics, Technical University of Sofia, 1756 Sofia, Bulgaria

**Keywords:** blood, non-invasive monitoring, portable polarizing system, optoelectronic method, smartphone, Stokes vector

## Abstract

The development of non-invasive optoelectronic technologies for human blood monitoring is one of the important research areas for medicine. A critical analysis of optoelectronic methods of blood research and the micromechanical systems based on them is carried out in this article. A design realization of a polarizing portable system for non-invasive monitoring of hematocrit as one of the basic homeostatic constants of the human body containing information about the microphysical parameters of blood cells has been substantiated. A physical model of polarized radiation conversion in a video information system of laser sensing of a biological research object has been formed. Visual and quantitative differences in the spatial distribution of polarization parameters of the scattered radiation for the states of the body with different hematocrit levels have been revealed. A scheme of a multichannel imaging portable system, based on a smartphone using miniature optical and microelectronic components of information conversion for non-invasive monitoring of microphysical blood parameters, has been created. The system implements the principle of polarimetric blood photometry and a multiparametric analysis of the polarization properties of the laser radiation scattered by blood. The developed portable optoelectronic system, based on a smartphone, can be used for rapid blood diagnostics in disaster medicine and the presence of clinical contraindications to the formation of invasive tests. The proposed polarization-based approach is a promising automated alternative to traditional devices and systems for the research of microphysical blood parameters.

## 1. Introduction

The development of micromanufacturing technologies for portable optoelectronic systems and biosensors contributes to their application in medicine for solving diagnostic problems. At the same time, the widespread use of smartphones has determined their choice in the field of affordable biomedical services as miniature laboratory-class devices.

The portability of smartphone-based systems is achieved either by installing an additional optical attachment for the built-in video camera, or by developing mobile applications for image processing. Carrying out bioanalytical research based on smartphones is based on the integration of software, advanced optical schemes, and microprocessor data processing technologies [1,2,3].

Smartphone-based video information devices are widely used as sensor input and display devices in micromechanical systems (MEMS), analytical units of detection systems, and devices for the noninvasive research of biological tissues and media accessible from the outside [4,5,6,7]. The rapid quantitative and qualitative assessment of the status of biological samples, based on the analysis of their images, proves that the software environment of smartphone-based systems can be used to efficiently diagnose various diseases [1,2,8].

The capabilities of portable optoelectronic devices based on smartphones to minimize invasive procedures in endoscopy, microscopy, and surgery, are being actively explored [2,4,5,9,10]. There are many researches using smartphones in polarimetric schemes as a miniaturized cost-effective visualization tool in various bioanalytical researches [1,2,3,10,11].

One of the important scientific directions in research for medicine is the development of non-invasive optoelectronic technologies for monitoring the state of human blood [12,13,14,15,16,17,18]. Modern medical diagnostics of diseases are traditionally carried out by performing a general blood test, which provides complete information about its microphysical parameters (such as, for example, the concentration of blood cells or their size distribution) [11,19,20]. At the same time, the concentration of insoluble blood cells linearly depends on blood hematocrit, whose increase or decrease may indicate the presence of cardiovascular diseases in the patient.

The use of devices based on invasive methods has limitations in critical clinical situations, during complex surgeries, and in the daily life of patients suffering from severe complications of cardiovascular diseases. In addition, they should not be used in emergencies when triaging and assisting casualties when a rapid (within 1–2 min) and qualitative assessment of the patient’s condition is required. These limitations are associated with the complexity of ensuring mobility, automation, and the continuity of the monitoring of blood microphysical parameters [19].

The great diagnostic significance of the blood test procedure, and its frequent repetition in medical institutions, makes it necessary to develop new methods and portable automated systems based on them. They let the attending physician receive objective information about the parameters of the patient’s blood in real-time (without its preliminary processing) and make prompt decisions.

In this regard, the following requirements are imposed on portable optical-electronic systems for the non-invasive monitoring of microphysical parameters of blood: efficiency; high accuracy; the ability to automate the process; to take into account the individual’s characteristics; and the use of the system in patients with relative (medication, previous diseases) and absolute (diseases of the cardiovascular system, respiratory organs, digestion) contraindications, where invasive blood sampling is prohibited.

The capabilities of optical-electronic and, in particular, polarization technologies in the field of diagnosing microphysical parameters of blood remain insufficiently researched. Therefore, it seems relevant to research and develop miniature polarization-optical devices, which, in combination with the capabilities of smartphones, will allow for the non-invasive automated monitoring of the patient’s blood condition in a portable mode.

Thus, the purpose of this work is to research optoelectronic, in particular polarization methods, for measuring the microphysical parameters of blood; to implement a portable video information system of the Stokes polarimeter based on modern micro-optical and microprocessor elements for the non-invasive monitoring of blood hematocrit, which is a stable constant of homeostasis and contains information about the anisotropic properties of blood cells.

## 2. Theoretical Aspects

It is known that blood is a light-scattering and absorbing medium. Therefore, to quickly determine its parameters, it is natural to use optical photometric methods, which allow for the quantitative analysis of substances without a distorting effect on their properties, and are the most acceptable in terms of accuracy and automation [21,22].

Whole blood consists of plasma (55% by volume) and formed elements (45% by volume), 99% of which are erythrocytes (hemoglobin-containing elements) and 1% are leukocytes and platelets [23,24]. Under normal physiological conditions (erythrocytes are not deformable), the optics of whole blood, as a highly concentrated turbid medium, is determined by the properties of erythrocytes and plasma. Concentrations of other blood-forming elements are negligible. Therefore, their influence on the parameters of radiation propagating in the blood can be neglected. The transmission of optical radiation by whole blood is affected by multiple scattering from erythrocytes and absorption by plasma. The scattering of photons by moving molecules and particles leads to the broadening and damping of the probing radiation. This should also be considered when conducting experiments for the correct interpretation of the results [20].

The percentage of erythrocytes and plasma in the total blood volume determines the hematocrit level. Since the concentration of erythrocytes linearly depends on the blood hematocrit (Equation (1)), research on the parameters of optical radiation scattered by blood will also make it possible to calculate the hematocrit [15,20]:

(1) $$\rho =\frac{HCT}{V_{e}},$$

where ρ is the concentration of erythrocytes, HCT is hematocrit, $V_{e}$ is the volume of an erythrocyte.

Tversky’s experimental theory is known, which considers the variation of the optical density of blood from the components of absorption and scattering of radiation by individual particles (erythrocytes) [20,25]. Based on this theory, there is a parabolic dependence of the intensity of light scattered by blood on erythrocyte concentration, which can also be compared with hematocrit levels in the blood. It was analytically shown that the absorption of light inside an erythrocyte is the same as in a hemoglobin solution, and a linear relationship was established between the absorption coefficient and blood hematocrit. The absorption coefficient of the blood layer can be expressed as [15,26]:

(2) $$\mu_{a}=\frac{HCT}{V_{e}}\cdot \sigma_{a},$$

where $\sigma_{a}$ is the optical absorption cross-section of a single particle.

For sufficiently small values of blood hematocrit (HCT < 0.2), the scattering coefficient is represented by the expression [15,27]:

(3) $$\mu_{s}=\frac{HCT\cdot \left(1-HCT\right)}{V_{e}}\cdot \sigma_{s},$$

where $\sigma_{s}$ is the optical scattering cross-section of an individual particle.

However, Equation (3) does not fully describe the dependence of the scattering coefficient on blood hematocrit, since it does not consider its physiological changes in tissues and cells, which also lead to changes in optical properties.

In the approximation of non-deformable hard spheres, the hematocrit level (HCT) cannot exceed 0.64 [20]. However, at values (HCT>0.5), erythrocytes become densely packed, and blood can be considered an almost homogeneous medium in which scattering particles containing hemoglobin and rounded by plasma are immersed. To eliminate errors in determining the concentration of erythrocytes due to their deformability, the following formula was obtained for the dependence of the scattering coefficient on the value of hematocrit (HCT) of thin blood layers [15,26]:

(4) $$\mu_{s}=\frac{HCT}{V_{e}}\cdot \sigma_{s}\cdot \left(1-HCT\right)\cdot \left(1.4-HCT\right).$$

The radiation scattering indicatrix is determined by the scattering anisotropy factor g, which is calculated as the average cosine of the scattering angle θ and is determined from the expression [15,26]:

(5) $$g=\langle \cos\theta \rangle =\overset{\pi }{\underset{0}{\int }}\rho \left(\theta \right)\cdot \cos\theta \cdot 2\pi \cdot \sin\theta d\theta$$

where ρ(θ) is the phase function approximated using the Henyi-Grishtein function [15,26]:

(6) ρ(θ)=14π·1−g2(1+g2−2·g·cosθ)32.

To consider the form of the scattering indicatrix, the reduced transport scattering coefficient is used, which is determined with Equation (7) [15,26]:

(7) $${\mu^{\prime }}_{s}=\mu_{s}\cdot \left(1-g\right).$$

In their article [15], the authors present the average values of light scattering parameters depending on blood hematocrit in the range from 0% to 70% at a constant shear rate of $500\;s^{–1}$. The graphs presented in the article [15] correspond to linearly increasing dependences of the absorption coefficient ($\mu_{a}$) and the reduced transport scattering coefficient (${\mu^{\prime }}_{s}$) on blood hematocrit (HCT). The scattering coefficient ($\mu_{s}$) increases linearly over the range of typical hematocrit values from 0% to 20% and does not change significantly with a further increase in blood hematocrit. The scattering anisotropy factor (g) remains practically unchanged and is in the range of 0.992–0.994 at typical hematocrit values from 0% to 10%. Further, with an increase in blood hematocrit, the scattering anisotropy factor (g) decreases to 0.975 at a typical hematocrit value HCT = 70% [15].

Thus, to research the clinically significant range of blood hematocrit from 30% to 70% using optoelectronic methods, it is necessary to consider the relationship between the absorption coefficient ($\mu_{a}$) and the reduced transport scattering coefficient (${\mu^{\prime }}_{s}$) of the probing radiation with hematocrit. To determine the prospects for the implementation of optical-electronic methods based on smartphones for monitoring blood hematocrit in vivo, it is necessary to review the methods existing in medicine and their comparative analysis.

## 3. Methods for Researching Blood Parameters

The most widely used methods are photometric non-invasive methods for researching the microphysical parameters of blood, which are implemented based on the optical sounding of the object under research through transmission [21,22] or on recording the spectral composition of scattered light [26,27,28,29].

A well-known method for researching blood hematocrit uses a two-spectrum radiation source [21,22]. The method is implemented through the correlation of the values of the two radiation fluxes that have passed through the finger, with the path length of the optical rays in the biological tissue. In this case, the light of one wavelength interacts with arterial vessels, and the other with soft tissues. As a result, photons reaching the photodetector after passing through an arterial vessel undergo pulsating modulation in intensity due to changes in the diameter of the vessel, while the signal from photons that have passed the arterial vessel remains constant during vessel pulsations.

The technical implementation of the spectral method was demonstrated in reference [27]. A device for the research of blood hematocrit using multispectral photometry of radiation backscattered by biological tissues of a human finger is proposed. The radiation from the halogen lamp passes through focusing optics and a multi-channel fiber bundle, which feeds the excitation radiation to the object of research (human finger). Then, the radiation scattered by the object of research is recorded by a dual-channel fiber-optic spectrometer. The spectrometer operates in the mode of measuring radiation kinetics at several wavelengths (up to six) in the visible and near-infrared regions of the spectrum simultaneously.

Through the first channel of the fiber-optic bundle, the object under research is excited. The distance radiation travels in biological tissue are approximately 0.26 to 2.10 mm and correspond to the intensity of diffuse scattering measured at six wavelengths: 0.66, 0.74, 0.81, 0.84, 0.91, and 0.95 µm, which is recorded through channels 2–9 of the fiber optic tourniquet.

The determination of blood hematocrit is carried out by calibrating the intensity of scattered radiation in various channels of the fiber optic bundle according to the values previously obtained through a standard certified method (empirically) for groups of patients with different levels of hematocrit.

The main advantage of these methods is the ability to ignore additional measurement errors that may be caused by the individual characteristics of the patient’s skin structure and surface condition. Significant disadvantages are the inability to consider the age and gender of the patient; the need for multiple measurements to ensure the specified accuracy.

Later, using the spectral method, an experiment was carried out in which the probing of the patient’s skin was used at various concentrations of hemoglobin in the blood and visualization of the reflection coefficient distribution [29]. The authors found that the reflection coefficient increases with the concentration of hemoglobin, and has a general tendency to decrease with an increasing wavelength of the probing radiation. The advantages of the method are in considering the individual characteristics of each patient; possibilities of application in endoscopic procedures; and the possibility of detecting differences in the size and concentration of healthy and diseased tissue cells. The disadvantages of the method are the strong impact of non-absorbing components of biological tissue on the optical coefficients. Furthermore, the authors based their conclusions on the assumption of the linear dependence of blood hemoglobin content on hematocrit, which is not always valid in some clinical situations. Therefore, this method does not allow for an accurate assessment of the hematocrit level.

The drawbacks of the method are considered in the currently developing polarization methods of blood diagnostics due to the high information content and sensitivity of the polarization characteristics of radiation to changes in the optical properties, geometry, and concentration of blood cells.

The effects of particle concentration and scattering anisotropy factor on the spot radius of light backscattered by subsurface structures of biological suspensions and turbid tissue phantoms are known [13,28]. The method is based on digital image processing of a light scattering circle detected on a charge-coupled device (CCD), using a crossed and parallel polarizer and analyzer scheme [19]. It was found that a change in the scattering coefficient $\mu_{s}$ does not change the azimuthal dependences, and the particle concentration affects the size of the output radiation region $r_{p}$. As the concentration of scattering centers increases by a factor of n, the size of the output radiation region $r_{p}$ also decreases by a factor of n [28].

Given that the depth of photon penetration into the medium is inversely proportional to the scattering coefficient, which in turn correlates with blood hematocrit (Formula (3)), it is possible to create a scattering profile for each blood vessel.

Polarization methods are often used in technical vision systems in medical practice [15,30,31,32,33,34]. It is known that the information carrier in a polarization image is both the values of the radiation intensity, but also the parameters of the state of its polarization, distributed over the cross-section of the beam scattered by the object. This makes it possible to perform a multi-parameter analysis of all spatial distributions to obtain a comprehensive picture of the anisotropic properties of the researched inhomogeneous medium.

The most common imaging method is “low-coherence polarization interferometry” [35]. To visualize the anisotropic structure of the subsurface layers of biological media, it is proposed to use the result of the interference of waves with given polarization states. Portable devices can be formed in this case both for reflection and scattering [11,28,35].

The method is based on the measurement and analysis of the elements of the Mueller matrix of an optically inhomogeneous medium, containing information about the anisotropy of the scattering and absorbing properties of the object of research.

The structure of such devices includes a block of the polarization state generator of the radiation probing the biological tissue, which consists of an optical radiation source and micro-optics (a polarizer and a quarter-wave phase plate). The reflected or scattered radiation from the sample enters the detector unit, which consists of a quarter-wave phase plate and an analyzer (to detect the polarized radiation component necessary for constructing the elements of the Mueller matrix of the bio medium), as well as a micro lens and a multi-element photodetector.

Advantages of the method: in some cases, it is non-invasive; there is the possibility of continuous monitoring and a high level of information content; it is possible to assess the state of individual organs by determining the state of blood plasma; and it takes into account the dependence of the measurement result on the density and thickness of cells, surface irregularities and the depth of light penetration, which makes it possible to obtain complete information about the object of research. Disadvantages of the method are as follows: lengthy measurement process; the difficulty of automating the measurement of the Mueller matrix; and it works only on open surfaces.

The analysis of partially polarized radiation at the output of an optically inhomogeneous medium can also be performed based on the measurement and visualization of the spatial distribution of the parameters of the Stokes vector [13,34,36]. In this case, the polarized light component will be due to the absorbing properties of blood plasma and will characterize its anisotropic properties, and the non-polarized component will be due to the scattering properties of blood erythrocytes, leading to depolarization of the input radiation.

Since the microphysical parameters of blood are related to the level of hematocrit, when it changes, the quantitative ratio between the intensities of the polarized and depolarized radiation components (that can be recorded and visualized) will also change. The Stokes vector method can be implemented non-invasively, with the possibility of automated measurements.

As a result of a critical review of optical-electronic methods for researching the microphysical parameters of blood, preference was given to polarization methods. The choice is justified by a sufficiently complete qualitative and quantitative picture of the distribution of light intensity after interaction with biological tissue, which makes it possible to implement a strategy for multiparametric analysis of changes in the anisotropic properties of blood for its diagnosis. At the same time, the absence of direct analogs reflects the prospects for implementing a new method for monitoring blood hematocrit based on Stokes polarimetry with visualization of the distributions of radiation parameters scattered by an object.

Thus, a hypothesis was put forward about the possibility of polarization monitoring of hematocrit based on the determination of the ratio between the absorbed and scattered components of the research.

## 4. Experimental Setup and Measurement Technique

**Figure 1:**
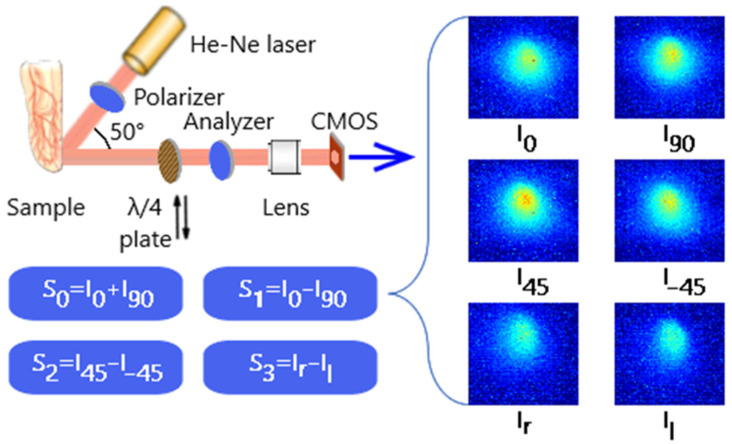
Scheme of the experimental setup and measurement technique.

The purpose of the experiments is to identify visual differences and quantify the distributions of polarization parameters in the physical simulation of bio-object states with different hematocrit levels. The experimental setup and measurement technique are shown in Figure 1.

The setup was formed according to the Stokes polarimeter scheme, with a sequential change in the parameters of the polarization elements of the receiving channel a finite number of times. The optical probing of a biological object was carried out through the nail plate of the little finger of the left hand of a person.

As a source of optical radiation, a 1 mW helium-neon (He-Ne) laser is used, which is commercially available and widely used in the diagnosis of human blood diseases [20]. The choice of the radiation wavelength is based on the analysis of the depth of penetration into the biological tissue of different spectral components.

It is known that in the ultraviolet (*λ* < 0.38 µm) and infrared (*λ* ≥ 2.0 µm) regions of the spectrum, outside the therapeutic window (0.6–1.6 µm), light penetrates the biological tissue for several cell layers or only a few millimeters, and in the short-wavelength region of the spectrum (0.38–0.40 µm), the penetration depth is from 0.5 to 2.5 mm. A further increase in the wavelength leads to the fact that the penetration depth of the visible (0.6–0.78 µm) and near infrared radiation (0.78–1.6 µm) increases to 8–10 mm. Since, for the research of blood through the nail plate, it is necessary to ensure a penetration depth into the biological tissue of at least 5 mm, a laser was chosen, the maximum spectral density of which falls on the red region of the spectrum (0.63 µm). In this case, the individual features of the thickness of the nail plate of the patient’s left little finger will not affect the measurement efficiency.

The choice of the power of the radiation source was carried out taking into account the safety of the patient and the absence of morphological changes in erythrocytes, which is necessary for the correct conduct of the experiment. It is known safety of the use of radiation with a wavelength of 0.63 µm and a power of 1.0 mW for intravenous laser blood irradiation with continuous exposure through the patient’s skin for an hour.

The power of laser radiation is chosen so that it does not cause the effect of increasing the degree of deformability of erythrocytes. This effect is extremely undesirable, since it leads to a change in the optical properties of the medium under research and a distortion of the sounding results. In addition, it cannot be taken into account for each patient, since the change in optical properties occurs individually for each patient.

The biological effect on the blood of laser radiation with a wavelength close to 0.633 µm and a power of more than 1.0–1.5 mW was also analyzed [20]. It was found that it is accompanied by the effect of increasing the degree of deformability of erythrocytes. This effect is extremely undesirable since it leads to a change in the optical properties of the medium under research and a distortion of the sounding results. In addition, it cannot be taken into account for each patient, since the change in optical properties occurs individually for each patient.

Thus, a helium-neon (He-Ne) laser with a wavelength of 0.633 µm and a power of 1 mW chosen as a source of optical radiation does not lead to a change in the structure of erythrocytes, and a person is not endangered by continuous exposure to laser radiation on the blood.

Four series of measurements of the polarization parameters of the output radiation were performed: the first series—at the initial state of human blood hematocrit and the next three series—at the changed (decreased) hematocrit states compared to the initial one.

When simulating a decrease in hematocrit, measurements were taken after 0.5 h, after 1.0 h, and after 1.5 h.

The effectiveness of monitoring changes in the anisotropic properties of blood with changes in erythrocyte concentration, and hematocrit level, therefore, depends on the angle of registration of scattered radiation.

Analysis of scattering indicatrix of low-intensity laser radiation in blood with different erythrocytes concentrations shows that the maximum difference between intensities of scattered radiation is observed in the vicinity of the angle of 50 degrees when scattered radiation of whole blood and blood diluted by 25 times [35]. The specified angle was implemented in the scheme of the preliminary experiment (Figure 1).

**Figure 2:**
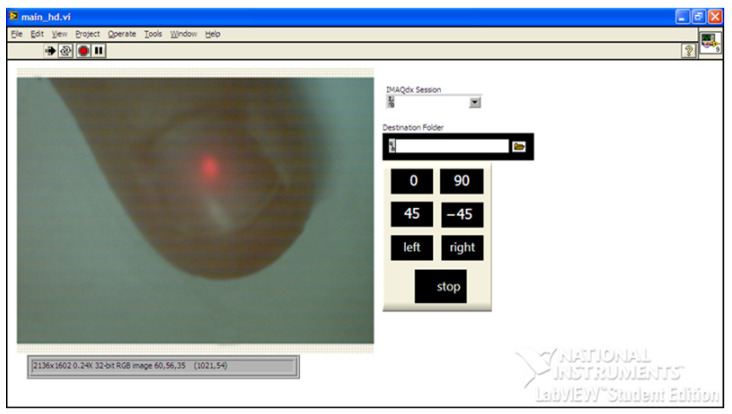
Software interface for recording the intensity distribution of angled scattered radiation on a matrix photodetector.

The measurement scheme consists of transmitting and receiving units. The transmitting unit includes a source of optical radiation (He-Ne laser) with a wavelength of 0.633 μm and a polarizer. The radiation scattered by the biological object enters the receiving channel and passes through the analyzer filter to identify the polarized components of the electric field strength vector in the output beam, which are necessary for the formation of the Stokes vector. Next, the focusing optical system (lens) forms an image of the scattering spot on a matrix photodetector Complementary Metal-Oxide-Semiconductor (CMOS) (Figure 2).

The registration and primary processing of the coordinate distributions of irradiance on a matrix photodetector were carried out using the software developed in the LabVIEW (12.0.0, National Instruments, Austin, Texas, USA) environment, the main window of which is shown in Figure 2.

The program allows you to read the irradiance values of each pixel and save them to the selected directory separately for the red, green, and blue channels of a video camera based on a color matrix photodetector. The names of saved text files $I_{0}$, $I_{90}$, $I_{45}$, $I_{–45}$, $I_{r}$, $I_{l}$, corresponding to the current position of the axes of the polarizing elements, and data files are generated by pressing the corresponding buttons of the main window (Figure 2).

During the first four measurements, the direction of the analyzer transmission axis changes relative to the fixed polarizer, which sets the initial polarization azimuth to the probing radiation; during the last two measurements, a phase-shifting element is introduced into the path of the rays scattered by the sample to record the circularly polarized components of the output radiation (Figure 1).

The files obtained as a result of the experiment are further processed using the developed script written in the MATLAB software (MATLAB 9.7, MathWorks, Natick, Massachusetts, USA). The program reads files from folders and calculates the parameters of the Stokes vector $S_{0}$, $S_{1}$, $S_{2}$, $S_{3}$ for each image pixel according to the matrix expression [34,36]:

(8) $$S=\left[\begin{array}{c} S_{0}\\ S_{1}\\ \begin{array}{c} S_{2}\\ S_{3} \end{array} \end{array}\right]=\left[\begin{array}{c} I_{0}+I_{90}\\ I_{0}-I_{90}\\ \begin{array}{c} I_{45}-I_{–45}\\ I_{r}-I_{l} \end{array} \end{array}\right],$$

where $I_{k}$ is signal values: *I*_0_ at a matched position of the polarizer and analyzer, $I_{90}$ at a crossed position, $I_{45}$ when the transmission axes of the polarizer and analyzer are at an angle of 45 degrees, $I_{l}$ with a similar position of the axes and the presence of a *λ*/4 plate in the scheme, $I_{–45}$ when the transmission axes of the polarizer and analyzer are at an angle of –45 degrees, $I_{r}$ with a similar position of the axes and the presence of *λ*/4 plate in the scheme.

Knowing the parameters of the Stokes vector makes it possible to carry out a complex polarization analysis of the beam of rays leaving the system, as well as to determine the residual degree of polarization of the input radiation according to the formula [34,36]:

(9) $$P=\frac{\sqrt{S_{1}{}^{2}+S_{2}{}^{2}+S_{3}{}^{2}}}{S_{0}}.$$

Azimuth α and ellipticity β of the ellipse of the polarization state of the output radiation are calculated, respectively, by the formulas [34,36]:

(10) $$\alpha =\frac{1}{2}\cdot arctg\left(\frac{S_{2}}{S_{1}}\right);\;$$

(11) $$\beta =tg\left(\frac{1}{2}\cdot arcsin\left[\frac{S_{3}}{\sqrt{S_{1}{}^{2}+S_{2}{}^{2}+S_{3}{}^{2}}}\right]\right).$$

These actions were performed for the red channel of the video camera since the maximum spectral density of the optical radiation source falls on the red region of the spectrum.

For each series of the experiment, six files were recorded with information on the spatial distribution of intensity in the cross-section of the radiation scattered by the sample for each of the simulated states of the body, guaranteed to differ from each other in the hematocrit level.

## 5. Experimental Results

In the digital processing of images, it is necessary to take into account possible displacements of a biological object and elements of the installation, which can occur during measurements and essentially influence the reliability of results. In this connection, all polarization images were combined according to the energy center of the first image.

An algorithm was used to estimate the coordinates of the energy center of the image of a point object according to the formulas:

(12) $$X=\frac{\sum_{i=1}^{M}\left[n_{i}\cdot Q_{s}\left(n_{i}\right)\right]}{\sum_{i=1}^{M}Q_{s}\left(n_{i}\right)};Y=\frac{\sum_{j=1}^{N}\left[m_{j}\cdot Q_{s}\left(m_{j}\right)\right]}{\sum_{j=1}^{N}Q_{s}\left(m_{j}\right)},$$

where $Q_{s}\left(n_{i}\right)$ and $Q_{s}\left(m_{j}\right)$ are the total signals resulting from the addition of signals from a small group of elements of the i-th column and the j-th row of the photodetector in the vicinity of the element of the highest signal, N and M are the number of columns and rows of the matrix, respectively, $n_{i}$ and $m_{j}$ are numbers corresponding to the coordinates of elements along the lines and columns of the photodetector.

The distribution of illumination along the *OX* axis created by the optical system from a point source corresponds to the charge relief $Q\left(X_{i}\right)$ along the surface of the multielement structure of the photodetector, as a function of the spatial coordinate of the element positions and often has the form of Gaussian distribution. It is known that in the case of a lens optical system for small (up to a few degrees) angles of incidence of the rays on the plane of the entrance pupil, the weight function of the lens can be approximated by a Gaussian rotation:

(13) E(x,y)=(Fc2πR2)·e−(n−n0)2+(m−m0)22πR2,

where R is the conditional radius of the scattering circle.

A program code was developed in the MATLAB software (MATLAB 9.7, MathWorks, Natick, Massachusetts, USA) environment to calculate the coordinates of energy centers of six images corresponding to the radiation intensity distributions $I_{0}$, $I_{90}$, $I_{45}$, $I_{–45}$, $I_{r}$, $I_{l}$ at different states of the polarization filter of the receiving channel of the setup and their matching was performed. Then, using Formula (8), the components of the total Stokes vector of radiation at the output of the object under research were calculated pixel by pixel. Using Formulas (9)–(11), a similar calculation was performed for the distributions of the degree of polarization, azimuth, and ellipticity of the radiation scattered by biological tissues.

**Figure 3:**
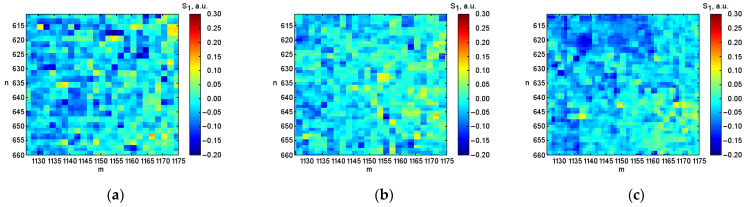

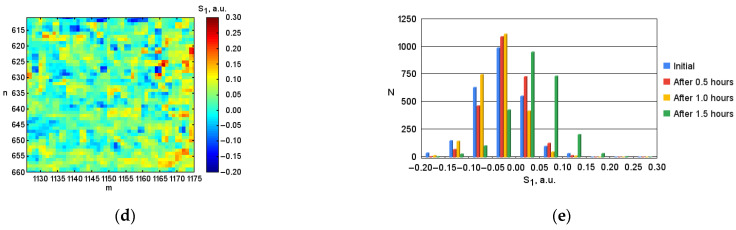
Coordinate distributions of the parameter of the Stokes vector $S_{1}$, where: (**a**) is the initial hematocrit level; (**b**) is the hematocrit level after 0.5 h; (**c**) is after 1.0 h; (**d**) is after 1.5 h, and (**e**) the corresponding histograms of the object under investigation.

**Figure 4:**
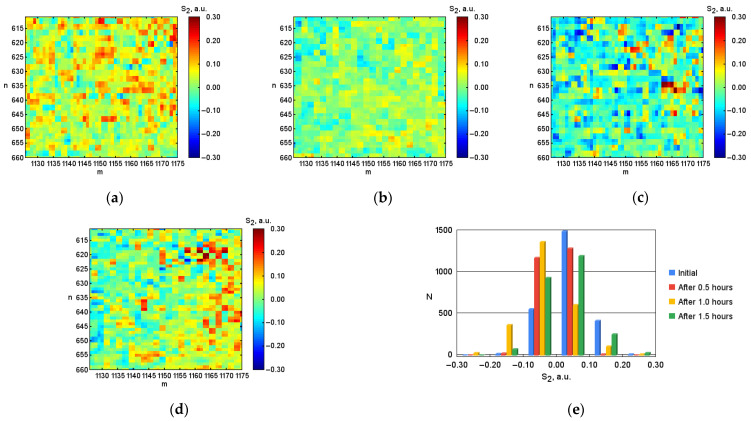
Coordinate distributions of the parameter of the Stokes vector $S_{2}$, where: (**a**) is the initial hematocrit level; (**b**) is the hematocrit level after 0.5 h; (**c**) is after 1.0 h; (**d**) is after 1.5 h, and (**e**) the corresponding histograms of the object under investigation.

**Figure 5:**
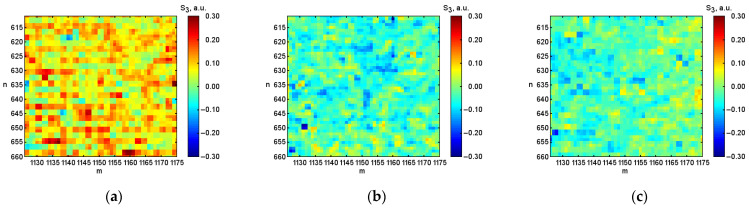

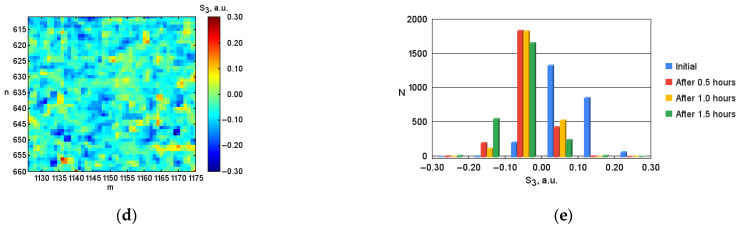
Coordinate distributions of the parameter of the Stokes vector $S_{3}$, where: (**a**) is the initial hematocrit level; (**b**) is the hematocrit level after 0.5 h; (**c**) is after 1.0 h; (**d**) is after 1.5 h, and (**e**) the corresponding histograms of the object under investigation.

**Figure 6:**
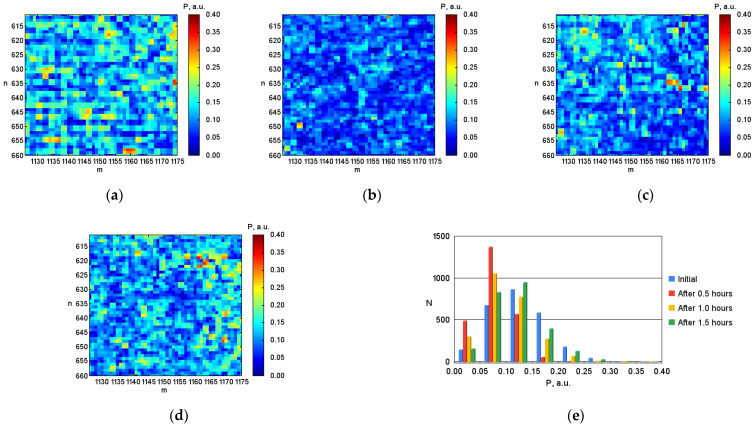
Coordinate distributions of the degree of polarization P, where: (**a**) is the initial hematocrit level; (**b**) is the hematocrit level after 0.5 h; (**c**) is after 1.0 h; (**d**) is after 1.5 h, and (**e**) the corresponding histograms of the object under investigation.

**Figure 7:**
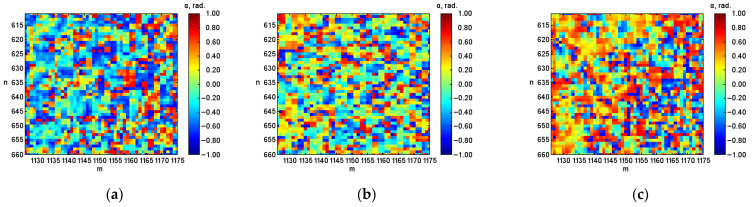

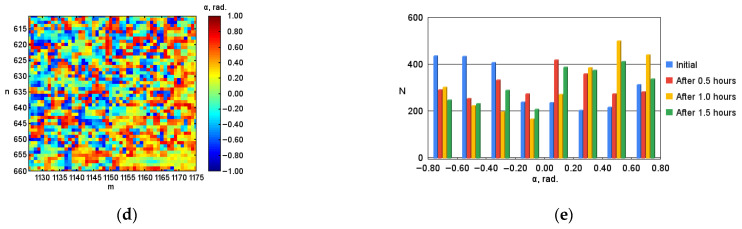
Coordinate distributions of the polarization ellipse (azimuth) α, where: (**a**) is the initial hematocrit level; (**b**) is the hematocrit level after 0.5 h; (**c**) is after 1.0 h; (**d**) is after 1.5 h, and (**e**) the corresponding histograms of the object under investigation.

**Figure 8:**
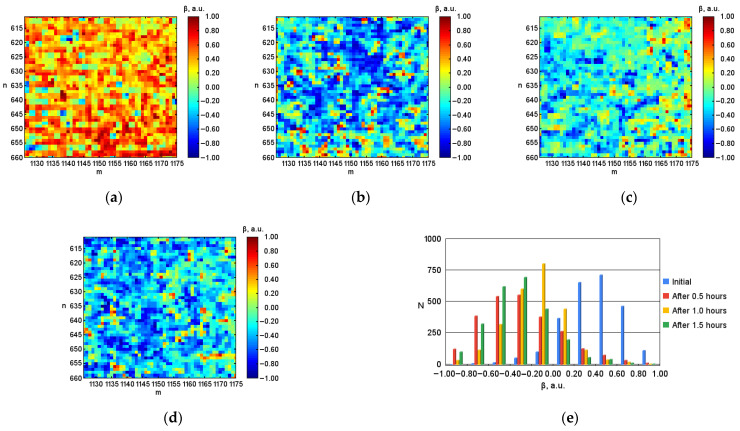
Coordinate distributions of the polarization (ellipticity) ellipse β, where: (**a**) is the initial hematocrit level; (**b**) is the hematocrit level after 0.5 h; (**c**) is after 1.0 h; (**d**) is after 1.5 h, and (**e**) the corresponding histograms of the object under investigation.

After digital image processing, the visualization of the coordinate distributions of the Stokes vector parameters $S_{1}$, $S_{2}$, $S_{3}$ (Figure 3, Figure 4 and Figure 5), the polarization state parameters P, α*,* β (Figure 6, Figure 7 and Figure 8) of the output radiation for four series of the experiment and constructed their corresponding histograms were performed. The figures show a sample (50 × 50) of the proximity of the found combined energy center of the spatial images of the radiation intensity distributions.

The average values of the parameters of the Stokes vector in conventional units, and the parameters of the state of polarization of the output radiation, are presented in Table 1.

The boundaries of error intervals for pixel-by-pixel measurement of Stokes vector parameters ($\delta_{S_{1}},\delta_{S_{2}},\delta_{S_{3}}$), degrees of polarization ($\delta_{P}$), azimuth ($\delta_{\alpha }$), and ellipticity of the radiation ellipse ($\delta_{\beta }$) with a confidence probability of 0.95, were calculated using the method of statistical processing of indirect measurements of the polarization characteristics of laser radiation [37].

The following error bounds were obtained for the Stokes vector parameter measurements ($\delta_{S_{1}}=\pm 8$%, $\delta_{S_{2}}=\pm 10$%, $\delta_{S_{3}}=\pm 8$%), polarization degrees ($\delta_{P}=\pm 12$%), azimuth ($\delta_{\alpha }=\pm 29$%) and ellipticity of the emission ellipse ($\delta_{\beta }=\pm 2$%).

The obtained boundaries of the intervals of relative errors in measuring the parameters of the Stokes vector, the degree of polarization, azimuth, and ellipticity of the radiation are quite high. This is primarily because for each series of the experiment, the measurements were carried out sequentially over a certain period, within which natural movements of the flows of the shaped elements in the vessels, physiological changes in the blood, and weak mechanical movements of the object are possible. These phenomena lead to the appearance of additional errors, which are difficult to consider analytically.

In addition, the boundaries of the intervals of relative errors for measuring the parameters of the Stokes vector, the degree of polarization, azimuth, and ellipticity of the radiation were obtained without using any calibration technique. In this regard, when implementing a smartphone-based portable polarization system for monitoring microphysical parameters of blood, the use of calibration will make it possible to introduce appropriate corrections into the single instrumental matrix of the receiving channel of the developed system. This will make it possible to reduce the systematic error introduced by real scheme elements.

## 6. Discussion of the Results

An analysis of the coordinate distributions of the polarization parameters of the output radiation (Figure 3–8), and the numerical values presented in the Table 1, made it possible to establish that there are significant visual differences for the simulated conditions of the body with different levels of hematocrit. At the same time, sharper changes are observed for the parameters of the polarization ellipse. In particular, for the ellipticity of radiation, this is demonstrated by noticeable changes in the color palette on the coordinate distributions from red at the initial blood hematocrit (Figure 8a) to blue at the hematocrit level after 1.5 h (Figure 8d). In addition, the change in the color palette also confirms the obtained experimental numerical values for ellipticity, presented in the table, which change from 0.389 at the initial hematocrit level, to –0.328 at the hematocrit level after 1.5 h.

In the physical modeling of a decrease in the concentration of erythrocytes (decrease in hematocrit) at certain intervals (0.5 h and 1.0 h), the degree of polarization increases compared to the initial state. However, the obtained experimental numerical values presented in the table demonstrate a slight decrease in the degree of radiation polarization from 0.128 at the initial hematocrit level to 0.101 at the hematocrit level after 1.0 h. The confirmation of the obtained experimental numerical values also demonstrates a change in the color palette on the coordinate distributions of the degree of radiation polarization from blue-green at the initial hematocrit level (Figure 6a) to blue at the hematocrit level after 1.0 h (Figure 6c).

With the gradual stabilization of the hematocrit level (after 1.5 h), over time, the numerical value of the degree of polarization again takes a value close to the original (at the initial hematocrit level). This conclusion is also confirmed by the obtained experimental numerical values presented in the table for the degree of radiation polarization, as well as the change in the color palette on the coordinate distributions for the degree of radiation polarization.

A return to the original value is also characteristic of the parameters of the polarization ellipse, in particular, for the azimuth. This is also demonstrated by noticeable gradual changes in the color palette on the coordinate distributions from the predominantly blue color at the initial hematocrit level (Figure 7a) to the predominant yellow color at the hematocrit level after 0.5 h and after 1.0 h (Figure 7b,c) to predominantly blue at hematocrit after 1.5 h (Figure 7d). In addition, the return to the original value for the azimuth is shown by the obtained experimental numerical values presented in the table, which change from –0.105 at the initial hematocrit level to 0.077 at the hematocrit level after 1.5 h.

The same conclusions can be drawn by analyzing the histograms of the corresponding parameters. For example, for the parameter ellipticity of the polarization ellipse (Figure 8e) there is a shift of the bar diagram from the maximum average value lying between 0.40 and 0.60 at the initial hematocrit level (blue color) to the left to the maximum average value lying between –0.40 and –0.20 at the hematocrit level after 1.5 h (green color).

The conclusion made about the gradual return of the numerical values of the polarization degree parameter from the initial hematocrit level to the hematocrit level after 1.5 h is confirmed by the analysis of the histograms obtained for the parameter under research (Figure 6e). There is a shift of the bar diagram from the maximum mean value lying between 0.10 and 0.15 at baseline hematocrit level (blue color) to the left to the maximum mean value lying between 0.05 and 0.10 at hematocrit level after 1.0 h (yellow color). Then, when the hematocrit level stabilizes (after 1.5 h), the bar diagram returns to its original position (shifts to the right), and the maximum average value takes on a value close to the value at the initial hematocrit level, lying between 0.10 and 0.15 (green color).

The return to the initial value is also characteristic of the parameter of the azimuth of the polarization ellipse of the output radiation. An analysis of the bar diagrams for the researched parameter (Figure 7e) allowed for establishing that numerical values for the initial level of hematocrit (blue color) and the level of hematocrit after 1.5 h (green color) are symmetrical relative to 0.00. Thus, when averaging the numerical values for the considered conditions, the average value will lie in the vicinity of zero, which indicates the return of the parameter under research to the initial value.

Thus, the most informative parameters that reflect the actual return to the original experimental numerical values with gradual blood stabilization are the parameters of the degree of radiation polarization and the parameter of the polarization ellipse, in particular, the azimuth.

The correct interpretation of these changes will certainly require repeated series of experiments, and most importantly, invasive hematocrit monitoring, as the most accurate research method.

The results obtained during the experimental research confirm the hypothesis of the possibility of the non-invasive polarization monitoring of hematocrit based on determining the ratio between the absorbed and dispersed components of the research in the blood.

## 7. Structure and Operating Principle of the System

The visual Stokes polarimeter was chosen as an analog for the development of the optical scheme of the portable polarization system. The requirement of portability, automation, and speed, will be met by the simultaneous measurement of the full set of radiation polarization parameters [38].

**Figure 9:**
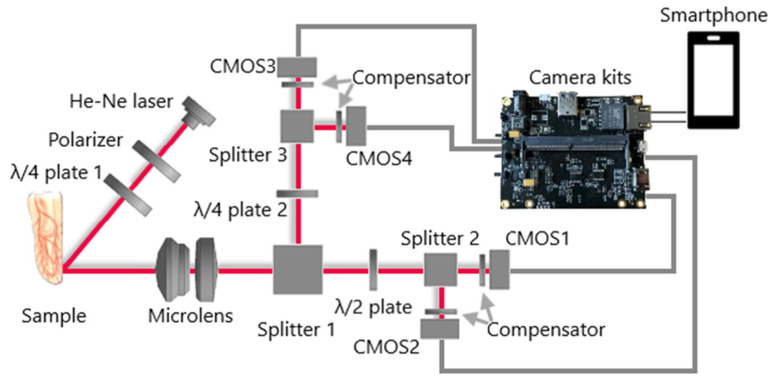
Structure of a polarization system based on a smartphone.

Figure 9 shows a diagram of a portable polarization system connected to a smartphone to visualize the distribution of polarization states of radiation scattered by a sample. The system consists of a transmitter and a four-channel receiver.

The transmitting unit contains a source of optical radiation (He-Ne laser) and miniature optical elements (polarizer and *λ*/4 plate 1) for the formation of a given state of polarization of radiation probing biological tissue. The channels of the receiving unit contain optical elements (micro lens; three splitters; *λ*/4 plate 2; *λ*/2 plate; compensators) for the formation and registration of four irradiance distributions on four matrix photodetectors (CMOS1-4). The scheme implements the following orientation of the anisotropic axes of the polarizing elements: the fast anisotropic axis *λ*/2 plate is oriented at an angle of 22.5 degrees, and the axis *λ*/4 plate 2 is oriented at an angle of 45 degrees to the selected plane containing the direction of beam propagation (plane of reference).

A low-power laser module with a wavelength of 0.635±0.005 µm and a power of 1 mW is used as a source for coherent optical research. The exposure of the blood to radiation with the above parameters does not lead to changes in its structure [39].

The receiving unit of the polarization system is formed in such a way that the length of the optical path of the radiation scattered by the sample from the receiving micro lens to the sensitive areas of the matrix receivers of each channel is the same. In this case, all four images of the radiation scattering circle with the sample surface have the same magnification for each channel over the entire field of view of the matrix, which makes it possible to simultaneously record four images without aberrations for their further digital processing.

Digital processing of polarization images is performed in the electronic unit (camera kits). To visualize the results, an information display device (smartphone) is used, the LI-NANO-CB-POE module (LI-NANO-CB-POE-IMX185CS-Q, Fremont, California, USA) is used as an electronic image processing unit, which ensures efficient operation with four miniature video cameras (LI-IMX185-MIPI-CS, Fremont, California, USA) based on color matrix photodetectors in conditions of size and weight restrictions. Camera kits were developed by Leopard Imaging (Fremont, California, USA) and can be used on the Jetson Nano/Xavier NX platform (NVidia, Santa Clara, California, USA) with the ability to connect cameras via Power over Ethernet (PoE). Due to the camera kits used, the required portability of a full-fledged smartphone-based polarization system is ensured. Data transfer from the camera kits to a smartphone can be carried out either via a USB Type-A-USB Type-C cable or via a universal Wi-Fi adapter, which is connected to the camera kits USB socket and wirelessly connected to a smartphone.

Thus, a portable polarization system based on a smartphone for monitoring human blood hematocrit has been developed, and can be technically implemented and used in emergencies for the timely detection of somatic abnormalities and shifts of this indicator from the norm, that in turn will allow for correction of blood hyper viscosity at an early stage and the prevention of the development of severe complications of diseases leading to the death of the patient.

## 8. Conclusions

The research studies the prospects for the implementation of a new approach to the research of the microphysical parameters of the blood. An approach to non-invasive monitoring of blood hematocrit is proposed, which consists of probing the blood-containing tissue through the nail plate of the little finger of the left hand of a person with polarized radiation, measuring the distribution of the intensity of the radiation scattered at an angle, and calculating the Stokes vector, which is used to calculate the most relevant parameters of the radiation polarization state (polarization degree and azimuth). The human blood hematocrit level is determined by comparing the results with empirical calibration characteristics obtained through a more accurate measurement method.

A portable smartphone-based four-channel polarization system that allows simultaneous measurement of the full set of Stokes vector parameters has been implemented. It processes four images of coordinate intensity distributions using a camera kit. The results are displayed on the smartphone screen. The portable polarization system can be in demand in the medical field as a fast and automated alternative to traditional invasive devices and systems for real-time monitoring of human blood hematocrit.

## Figures and Tables

**Table 1:** Numerical values of the experimental results.

**Series**	Parameter	$S_{1}$	$S_{2}$	$S_{3}$	P	α, rad.	β
	Hematocrit						
1	Initial	–0.029	0.044	0.081	0.128	–0.105	0.389
2	After 0.5 h	–0.018	0.003	–0.039	0.080	0.007	–0.292
3	After 1.0 h	–0.037	–0.030	–0.029	0.101	0.116	–0.173
4	After 1.5 h	0.035	0.020	–0.062	0.118	0.077	–0.328

## Data Availability

Data are contained within the article.
